# The Cost of Provisions in Institutions.—IX

**Published:** 1904-06-18

**Authors:** 


### HOSPITAL ADMINISTRATION.

### CONSTRUCTION AND ECONOMICS.

### THE COST OF PROVISIONS IN INSTITUTIONS.—IX.

BY THE AUTHOR OF "HOSPITAL EXPENDITURE—THE COMMISSARIAT."

(*Continued from page* 195.)

CHARING CROSS HOSPITAL (150 AVAILABLE BEDS).

THE cost of provisions at this hospital during the last five years has been as follows:—

| Year | Number of occupied beds | Number of available beds | Average per occupied bed |
|---|---|---|---|
| | | | £ |
| 1902 ... | 132 | 150 | $27\frac{1}{4}$ |
| 1901 ... | 132 | 150 | $21\frac{3}{4}$* |
| 1900 ... | 147 | 175 | $24\frac{1}{4}$ |
| 1899 ... | 151 | 175 | $23\frac{3}{4}$ |
| 1898 ... | 141 | 175 | $24\frac{1}{4}$ |

* Hospital entirely closed for two months.

The extensive alterations which have been in progress at the hospital during the last two or three years have given rise to natural fluctuations in expenditure, and it will appear, on closer examination, that, so far from a wide discrepancy as between £$27\frac{1}{4}$ and £$21\frac{3}{4}$ per occupied bed in the years 1902 and 1901 respectively, the cost of provisions for those years is practically identical. The average number of occupied beds in 1902 and 1901 is stated to be identical, yet during 1901 the number of in-patients received was 1,584, as against 1,986 in 1902. The hospital was entirely closed in the former year for two months, and the average of occupied beds has been calculated in the published report on the basis of a 10-months' year. The following table will show this more clearly:—

| Year | Available beds | Average of occupied beds | Number of in-patients | Total cost of provisions |
|---|---|---|---|---|
| | | | | £ |
| 1902 ... | 150 | 132 | 1,986 | 3,700 |
| 1901 ... | 125* | 110* | 1,584 | 2,880 |

* Taking the whole year.

| Year | Estimated cost of patients at 11d. a day | Average cost of boarding in-patient | Average cost of provisions exclusive of patients: Per occupied bed | Average cost of provisions exclusive of patients: Per available bed |
|---|---|---|---|---|
| | £ | £ | £ | £ |
| 1902 ... | 2,000 | 1 | $12\frac{1}{2}$ | $11\frac{1}{4}$ |
| 1901 ... | 1,600 | 1 | $11\frac{1}{4}$ | $10\frac{1}{4}$ |

It is rather to be regretted that managers do not uniformly reckon the average of occupied beds on a basis of 12 months to the year, whether or no the hospital be closed for part of that time. It is something of an enigma to read that the number of occupied beds and the average stay of the patient are identical during periods showing a difference of 400 in the numbers received.

The numbers boarded "other than patients" at the Charing Cross Hospital are as follows:—

| — | Number | Average of occupied beds to each | Average of available beds to each |
|---|---|---|---|
| Medical officers... | 10 | 13·2 | 15 |
| Nurses ... ... | 51 | 2·5 | 3 |
| Female servants and ward-maids | 26 | 5 | 6 |
| Total ... ... | 87 | 1·5 | 1·6 |

The percentage of those boarded "other than patients" of the total number of inmates is 40.

The cost of the patient is estimated at about 11d. a day, or £16 a year.

The cost of the nursing staff is estimated to be between 6s. and 7s. a week, say about £17 a year.

The cost of the servants can hardly be less than about 6s. a week, or £16 a year.

Deducting at these rates for patients, nursing staff, and servants, there remains about 17s. or 18s. a week for the board of each of the medical officers, or from £42 to £44 a year.

As the system of accounts does not admit of the separation of the expenditure under the various headings enumerated, these estimates must be accepted with caution. The estimate of 11d. a day for the patients may easily, we think, be higher than what is actually expended.

Since the beginning of 1903 arrangements have been made to provide all the men-servants, porters, etc., with dinner in the hospital. This plan is now adopted in most institutions, the loss of time and other inconveniences resulting from sending the men to get their dinner outside being found to outbalance the extra trouble of providing an additional service of meals. The average cost for each item of provisions is shown in the following table:—

| | |
|---|---|
| Average daily number of patients ... ... ... | 132 |
| " " " non-patients ... ... | 87 |
| Total number boarded daily in institution ... | 219 |

| — | Total cost | Average per occupied bed | Average per head, all inmates |
|---|---|---|---|
| | £ | £ s. | |
| Meat ... ... | 1,306 | 9 17 | 6·0 |
| Fish, poultry, etc.... | 301 | 2 5 | 1·7 |
| Bacon, butter, and cheese ... ... | 471 | 3 11 | 2·3 |
| Eggs ... ... | 128 | 0 18 | 0·11 |
| Milk ... ... | 621 | 4 14 | 2·16 |
| Bread and flour ... | 274 | 2 1 | 1·5 |
| Grocery ... ... | 292 | 2 4 | 1·6 |
| Vegetables ... ... | 160 | 1 4 | 0·14 |
| Malt liquor ... | 96 | 0 14 | 0·8 |
| Ice and mineral waters ... | 49 | Patients only 0 7 | — |
| Wine and spirits ... | 114 | Patients only 0 17 | |

The amount of meat used daily for the patients is from 60 to 63 lbs., or an average of rather less than ½ lb. a head. This would include also meat for making broth and beef-tea. For the rest of the household, including, as we have seen, 87 persons, the average quantity of meat used is from 500 to 600 lbs. per week, or an average of from ¾ lb. to 1 lb. a head daily.

The quantity of milk required for the patients is 340 pints a day, or an average of about 2½ pints each.

In the household the quantity of milk used is about 40 pints a day, or less than ½ pint each; from which it is clear that milk is not used as a beverage.

Neither ice nor mineral waters are made in the hospital. The latter are presented as a free gift up to a certain quantity, and, if economy is to be effected by getting them made on the premises, it will not be denied that by this method economy itself is outdistanced.

The reconstruction of the basement, which has rendered possible the addition of new kitchen premises, has been of the greatest advantage to the commissariat department of the hospital. The new kitchen is large and airy, and has been fitted with the best apparatus for cooking by gas and steam. The walls are lined with ivory-white tiles, and the floor is paved with hard-pressed tiles of terra cotta. A scullery, two larders, two store-rooms, a housekeeper's room, and servants' hall have also been added, and all the rooms have been fitted with electric light. The importance of light, air, and space in the storage and preparation of food is now generally recognised in hospitals, and is leading to a far higher standard of cooking and of seemliness in the serving of the meals.

Theoretically such improvements might be expected to lower the average cost of provisions, since in the old-fashioned underground kitchens, where in some hospitals the pans of milk could be seen waiting in the dust and heat of midday till they could be carried off to the wards, there must have been much waste and deterioration in the food.

In practice we do not, however, know of any institution where new kitchens have resulted in lowered bills.

